# Homogeneous Liquid–Liquid Extraction of Rare Earths with the Betaine—Betainium Bis(trifluoromethylsulfonyl)imide Ionic Liquid System

**DOI:** 10.3390/ijms141121353

**Published:** 2013-10-28

**Authors:** Tom Vander Hoogerstraete, Bieke Onghena, Koen Binnemans

**Affiliations:** Department of Chemistry, KU Leuven, Celestijnenlaan 200F, P.O. Box 2404, Heverlee B-3001, Belgium; E-Mails: tom.vanderhoogerstraete@chem.kuleuven.be (T.V.H.); bieke.onghena@chem.kuleuven.be (B.O.)

**Keywords:** hydrometallurgy, ionic liquids, lanthanides, neodymium, phase transitions, rare earths, solvent extraction, thermomorphic behavior, UCST

## Abstract

Several fundamental extraction parameters such as the kinetics and loading were studied for a new type of metal solvent extraction system with ionic liquids. The binary mixture of the ionic liquid betainium bis(trifluoromethylsulfonyl)imide and water shows thermomorphic behavior with an upper critical solution temperature (UCST), which can be used to avoid the slower mass transfer due to the generally higher viscosity of ionic liquids. A less viscous homogeneous phase and mixing on a molecular scale are obtained when the mixture is heated up above 55 °C. The influence of the temperature, the heating and cooling times, were studied for the extraction of neodymium(III) with betaine. A plausible and equal extraction mechanism is proposed in bis(trifluoromethylsulfonyl)imide, nitrate, and chloride media. After stripping of the metals from the ionic liquid phase, a higher recovery of the ionic liquid was obtained by salting-out of the ionic liquid fraction lost by dissolution in the aqueous phase. The change of the upper critical solution temperature by the addition of HCl or betaine was investigated. In addition, the viscosity was measured below and above the UCST as a function of the temperature.

## 1. Introduction

Solvent extraction (SX) or liquid–liquid distribution is one of the major techniques applied for the separation and purification of metal streams [1]. The basis of this technique is the preferential distribution of a solute between two immiscible liquid phases. Generally, an aqueous solution containing the dissolved solute is brought into contact with another immiscible organic phase. The solute distributes between the two phases until equilibrium is reached. This equilibration is accelerated by strong agitation (stirring or shaking), as this will increase the phase boundary area between the organic and aqueous phase at which diffusion of the solute occurs.

Ionic liquids are organic salts that consist entirely of ions and have typically a melting point below 100 °C [2–4]. These types of compounds have recently gained a lot of interest because they have several interesting properties such as a negligible vapor pressure [5], a low flammability [6], a high thermal stability [7], and a broad electrochemical window and liquidus range [8]. Also, the tuneability of ionic liquids is one of their major advantages. Via a suitable choice of cation-anion combination, an ionic liquid with the appropriate properties for a given application can be obtained [9].

The beneficial properties of ionic liquids also gained the interest and curiosity of researchers in hydrometallurgy, searching for environmentally friendlier alternatives to volatile, flammable, and toxic organic solvents such as toluene, kerosene and chloroform which are applied in most solvent extraction systems [10–16]. Ionic liquids can be used as diluents in combination with traditional organic extracting agents. Another approach is incorporating a specific metal-coordinating group into their structure (task-specific ionic liquids or functionalized ionic liquids). It is evident that ionic liquids for use in solvent extraction systems should be immiscible with water. The ionic liquid can be made hydrophobic by choosing a cation with long alkyl chains (e.g., Cyphos^®^ IL 101 or Aliquat^®^ 336) [17,18]. However, in most cases hydrophobic fluorinated anions such as bis(trifluoromethylsulfonyl)imide (Tf_2_N^−^) or hexafluorophosphate (PF_6_^−^) are selected, because a larger variety of cations, with or without functionalities, can be selected for extraction of a particular metal. One of the disadvantages of using ionic liquids as diluents in extraction systems is their higher viscosity compared to molecular solvents [19,20]. For instance, 1-butyl-3-methylimidazolium bis(trifluoromethylsulfonyl)imide has a viscosity of 32 cP at 20 °C if it is water-saturated [21], whereas the viscosity of conventional organic solvents is about 1 cP. Therefore, intensive mixing, shaking, and heating up are required to increase the mass transfer and to obtain equilibrium in shorter time scales.

Some ionic liquids show thermomorphic behavior which means that they become miscible with any solvent above or below a certain temperature [22–33]. The upper critical solution temperature (UCST) of an ionic liquid is the temperature above which the ionic liquid is miscible with water in all proportions. Other ionic liquid systems have a lower critical solution temperature (LCST), defined as the temperature at which one homogeneous phase is formed below that temperature. Ionic liquids showing thermomorphic behavior with water are interesting for ionic liquid solvent extraction systems because mixing on molecular scale can be obtained just by heating or cooling the biphasic mixture until one homogeneous phase is obtained [17]. Viscosity is decreased and mass transfer and kinetics are accelerated in this way. This type of extraction is called homogeneous liquid–liquid extraction (HLLE), coalescence extraction or phase-transition extraction and has been used mainly for the extraction of natural products [34–36], although some examples of HLLE for metal extraction are also known [34,37,38]. To the best of our knowledge, the only example of HLLE for metals with ionic liquids has been reported by Vaezzadeh *et al.* [39]. They made use of the temperature-induced phase separation principle with the ionic liquid 1-hexyl-3-methylimidazolium tetrafluoroborate [C_6_mim][BF_4_] for the determination of silver via micro-extraction. The extraction was performed by cooling a 50 °C aqueous silver solution containing 4,4-bis(dimethylamino)-thiobenzophenone, NaPF_6_ and a small amount of [C_6_mim][BF_4_]. Phase separation was induced by a combination of decreasing the temperature and a salting-out effect with NaPF_6_.

**Figure 1:**
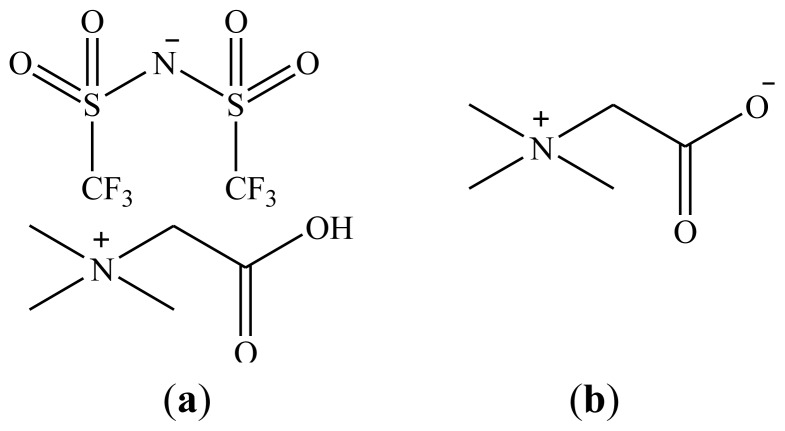
(**a**) Structure of the ionic liquid [Hbet][Tf_2_N]; and (**b**) The extracting agent betaine.

Recently, we communicated the proof-of-principle of HLLE of a large selection of metals (Ag, Cu, Zn, Fe, Co, Ni, In, Ga and several rare earths) with betaine as extractant and [Hbet][Tf_2_N] as organic phase (Figure 1) [40]. One homogeneous phase could be obtained by increasing the temperature of a 50 wt % ionic liquid mixture with water above the UCST of 55 °C and two phases were obtained again after cooling down, without the need for an extra salting-out agent. Stripping could be performed with HCl or HTf_2_N, in which a precursor for the ionic liquid [Hbet]Cl or the ionic liquid itself were obtained. In the present paper, several fundamental extraction parameters such as the kinetics (temperatures), loading, and a plausible extraction mechanism are given for HLLE of neodymium(III) with the [Hbet][Tf_2_N]–H_2_O system. Also, the influence of HCl or betaine on the UCST and the recovery of ionic liquid will be described.

## 2. Results and Discussion

### 2.1. Viscosity and Mass Transfer

The viscosity of [Hbet][Tf_2_N] at 60 °C is 351 cP [22], and thus significantly higher than that of conventional bis(trifluoromethylsulfonyl)imide ionic liquids used in solvent extraction systems [41]. This is due to the carboxylic group on the cation which forms an extra interaction with the anion via a hydrogen bond. [Hbet][Tf_2_N] takes up circa 13 wt % of water at room temperature and the viscosity of the water saturated ionic liquid at 23 °C is therefore 238 cP. It was observed that the viscosity increased if betaine was added to a water/ionic liquid mixture. After bringing 4 g of water-saturated ionic liquid in contact with 3.48 g of water containing 20 wt % of betaine, the viscosity increased to 582 cP at 23 °C. Ionic liquid extractions are often performed at higher temperatures, since there is an inverse relationship between the diffusion coefficient *D* and the viscosity η. The relationship can be described by the Stokes-Einstein equation:

(1) $$D=\frac{kT}{6\pi \eta r}$$

where *k* is Boltzmann’s constant, *T* is the absolute temperature and *r* is the radius of the molecule. A higher diffusion coefficient means that molecules are moving faster, that the contact time between different molecules is much shorter, and that extraction equilibrium is obtained faster. The viscosity decreased from 125 cP just below the UCST (40 °C) to 20 cP above the UCST at 65 °C.

The relationship between the viscosity and the temperature is given by the Arrhenius equation:

(2) $$\eta =\eta_{\infty }\cdot \text{exp}\;\left(\frac{E_{\text{a}}}{RT}\right)$$

where η_∞_ is the viscosity at infinite temperature and *R* is the universal gas constant. *E*_a_ is the activation energy, which is defined as the energy barrier which the ions must overcome to move in the mixture [42]. The equation is typically extended to other functions such as Vogel-Fulcher-Tammann (VFT) or Litovitz, which are a much better fitting of the viscosity-temperature dependence of ionic liquid [43–47]. The following equation can be obtained by taking the natural logarithm of Equation (2):

(3) $$\text{ln}\eta =\text{ln}\;(\eta_{\infty })+\left(\frac{E_{\text{a}}}{RT}\right)$$

**Figure 2:**
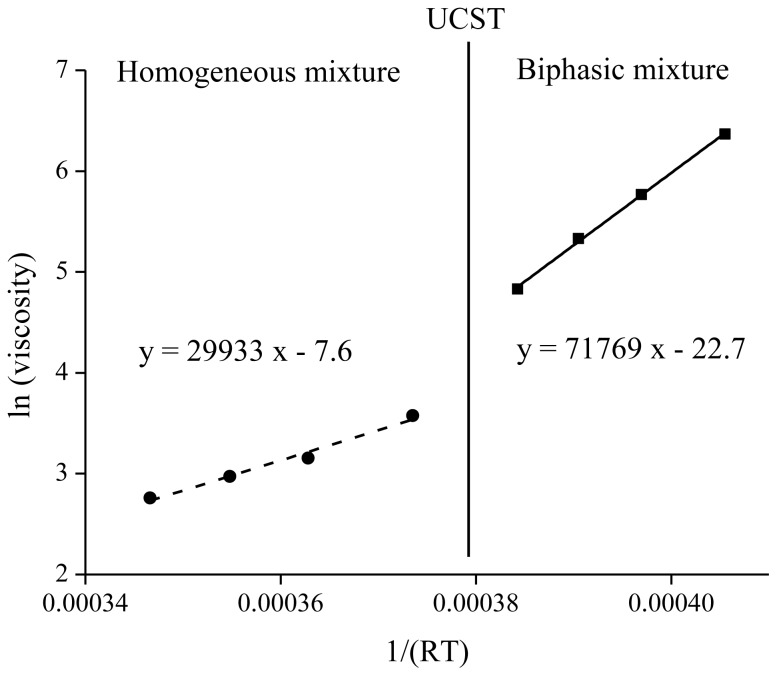
Arrhenius plot for [Hbet][Tf_2_N] in equilibrium above and under the upper critical solution temperature (UCST) with 53 wt % water containing the betaine extractant.

A linear fitting can be obtained for ln(η) as a function of 1/*RT*. The slope of the curve gives the activation energy *E*_a_ for the movements of the ions in the mixture. Four data points were obtained above and four below the UCST (Figure 2). An energy barrier of 71.7 kJ·mol^−1^ must be overcome to move ions in the ionic liquid below the UCST in biphasic mixture whereas an energy barrier of 29.9 kJ·mol^−1^ was obtained in the case of a homogeneous mixture. The energy necessary to move molecules along each other in the homogeneous state is almost 2.5 times lower than in the case of a biphasic mixture.

### 2.2. Distribution Ratios and Percentage Extractions

Distribution ratios (*D_M_*) are calculated by the following equation:

(4) $$D_{M}=\frac{{\left[M\right]}_{\text{org}}}{{\left[M\right]}_{\text{aq}}}$$

where [*M*]_org_ (mg/kg) and [*M*]_aq_ (mg/kg) are the metal concentration in the organic phase (*i.e.*, ionic liquid phase) and the aqueous phase, respectively. By measuring the metal concentration in the water phase after the extraction and taking into account the mass balance, Equation (4) can be rewritten:

(5) $${\left[M\right]}_{\text{org}}=\frac{{\left[M\right]}_{\text{aq},0}\cdot m_{\text{aq},0}-{\left[M\right]}_{\text{aq}}\cdot m_{\text{aq}}}{m_{\text{org}}}$$

where [*M*]_aq,0_ and [*M*]_aq_ are the metal concentrations in the aqueous phase before and after the extraction, respectively. *m*_aq_*_,_*_0_ and *m*_aq_ are the masses of the aqueous phase before and after the extraction, and *m*_org_ is the mass of the organic phase after extraction. Since the mass of both phases at the end of the extraction differs from the initial mass due to the water-ionic liquid mutual solubility, *m*_aq_ and *m*_org_ are unknown parameters. The initial masses of the aqueous and organic phase are used for these parameters as an approximation. Thus, it should also be noted that this calculated metal concentration in the organic phase is not completely correct due to the mass changes of the water and ionic liquid phase. This has also an effect on the distribution ratios and percentage extractions which are slightly overestimated. If the Total Reflection X-Ray Fluorescence (TXRF) results are consistent and seem reliable, it is recommended to use the measured organic metal concentration.

The percentage extraction (%*E*) of a metal is defined as:

(6) $$\%E=\frac{\text{Amount of extracted metal}}{\text{Total amount of metal}}\times 100\%$$

or

(7) $$\%E=\frac{{\left[M\right]}_{\text{org}}\cdot m_{\text{org}}}{{\left[M\right]}_{\text{org}}\cdot m_{\text{org}}+{\left[M\right]}_{\text{aq}}\cdot m_{\text{aq}}}\times 100\%$$

The initial mass of ionic liquid phase and aqueous feed are used as a good approximation for *m*_aq_ and *m*_org_, similar as for the calculation of the metal concentration in the organic phase.

The percentage stripping (%*S*) is defined as:

(8) $$\%S=\frac{\text{Amount of stripped material}}{\text{Total amount of metal}}\times 100\%$$

or

(9) $$\%S=\frac{{\left[M\right]}_{\text{aq}}\cdot m_{\text{aq}}}{{\left[M\right]}_{\text{org}}\cdot m_{\text{org}}+{\left[M\right]}_{\text{aq}}\cdot m_{\text{aq}}}\times 100\%$$

The masses of the different phases change during back-extraction, similar to the extraction step, and this change depends on the acid concentration used. Therefore, *m*_aq_ and *m*_org_ are unknown parameters in Equation (9), and they are approximated by the initial mass of the corresponding phase.

### 2.3. Extraction Parameters

Different operational parameters of the HLLE process were investigated. These include the heating time in the homogeneous phase, the temperature above UCST, the settling time, and settling temperature. In a recent communication [40], we proposed that the extraction of metals via homogeneous liquid–liquid extraction is faster than in the case of traditional extraction experiments, due to the mixing on molecular level above the UCST. To provide evidence for this hypothesis, the HLLE process has been compared with a conventional extraction process, and the influence of the heating time in the homogeneous phase above UCST on the extraction efficiency, was investigated.

**Figure 3:**
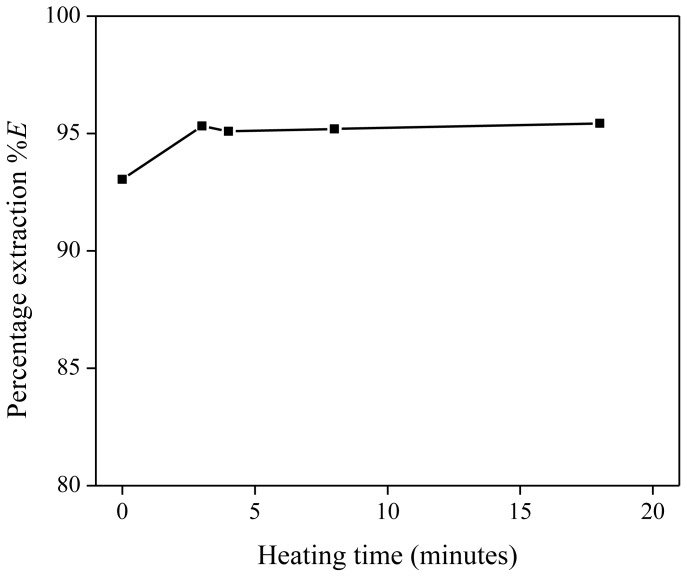
Percentage extraction (%*E*) as a function of the heating time (in minutes) above the UCST, for the extraction of Nd(Tf_2_N)_3_ in [Hbet][Tf_2_N]–H_2_O with betaine as extractant.

Five extraction mixtures with the same composition were prepared. Each solution contained 1 g of Nd(Tf_2_N)_3_ solution with a concentration of 1500 mg/kg Nd, 288 mg of betaine and 1 g of water-saturated [Hbet][Tf_2_N]. Four mixtures were heated at 60 °C during varying times (3 to 18 min) and stirred further at room temperature until a total stirring time of 20 min was reached. One extraction mixture was stirred at room temperature for 20 min and did not proceed via a homogeneous stage. This temperature is about 15 °C under the UCST as we will show later that 28.8 wt % of betaine decreases the UCST to below 40 °C (Figure 3). The lower percentage extraction for the heterogeneous extraction at room temperature confirms the slower extraction kinetics of heterogeneous mixing. Once the homogeneous stage had been reached, no significant changes in the percentage extraction (%*E*) were observed as a function of the time.

**Figure 4:**
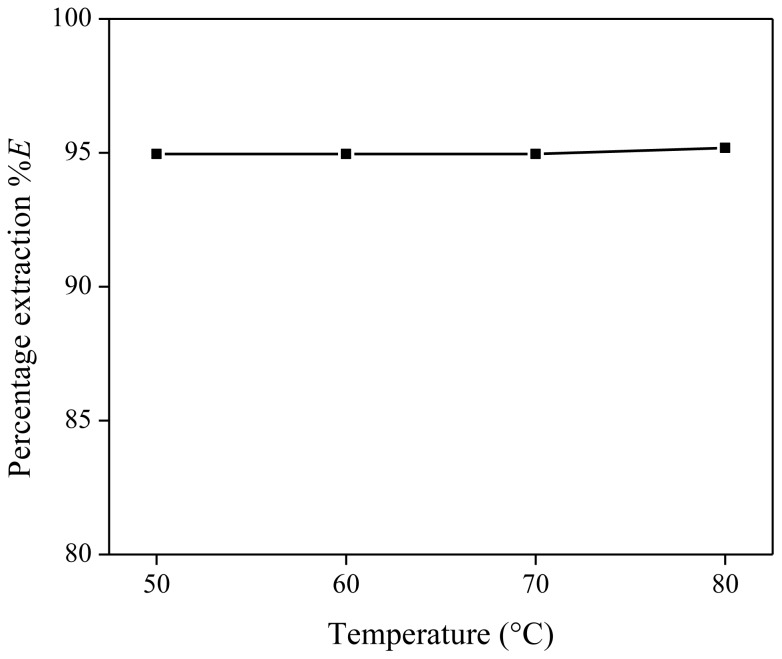
Percentage extraction (%*E*) as a function of the temperature of the homogeneous stage for the extraction of Nd(Tf_2_N)_3_ in [Hbet][Tf_2_N]–H_2_O with betaine as extractant.

Next, the influence of the temperature of the homogeneous stage was investigated by performing the same extraction four times, each time at a different temperature (50, 60, 70 and 80 °C). Each extraction mixture contained 1 g of [Hbet][Tf_2_N] and 1 g of the aqueous starting solution (890 mg/kg Nd with 20 wt % of betaine). For each extraction experiment, the distribution ratio was calculated and plotted *vs.* the temperature of the homogeneous stage (Figure 4). From these results, it is clear that the heating temperature during the homogeneous stage has no influence on the extraction equilibrium and the percentage extraction.

**Figure 5:**
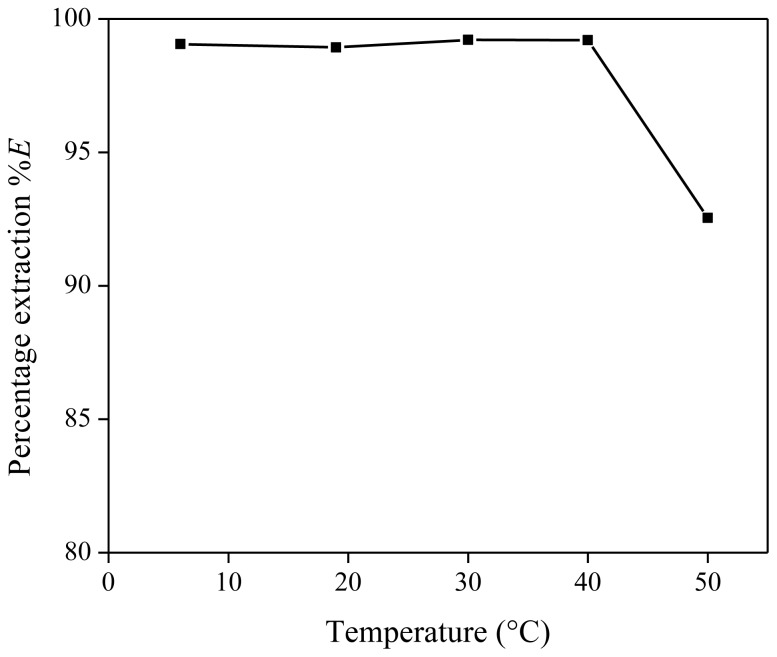
Percentage extraction (%*E*) as a function of the temperature to which the extraction mixture is cooled after the homogeneous stage for the extraction of Nd(Tf_2_N)_3_ in [Hbet][Tf_2_N]–H_2_O with zwitterionic betaine as extractant.

After the formation of one homogeneous phase and reaching the extraction equilibrium, the mixture is cooled to room temperature to reform the two-phase system. This is the so-called settling stage. Since the mutual solubility of [Hbet][Tf_2_N] and water is temperature dependent, the temperature to which the extraction mixtures are cooled during the settling stage of the extraction process, may have an influence on the extraction percentage of the metal ions. Different settling temperatures were tested, ranging from 6 to 50 °C. Each extraction mixture contained 1 g of [Hbet][Tf_2_N], 1 g of Nd(Tf_2_N)_3_ solution (1500 mg/kg Nd) and 200 mg of betaine (16.7 wt %). The percentage extraction showed constant values over the measured temperature range, except for the data collected at 50 °C, close to the UCST (Figure 5). This is because the aqueous and ionic liquid phases have a nearly similar composition at this temperature. We will show later on that the UCST of the [Hbet][Tf_2_N]–water mixture (16.7 wt % betaine) is just above 50 °C. Room temperature is therefore ideal to reinstall the two-phase equilibrium when cooling the extraction mixture during the settling stage. Small deviations at temperatures around 20 °C will not result in large differences in distribution ratios or percentage extraction.

**Figure 6:**
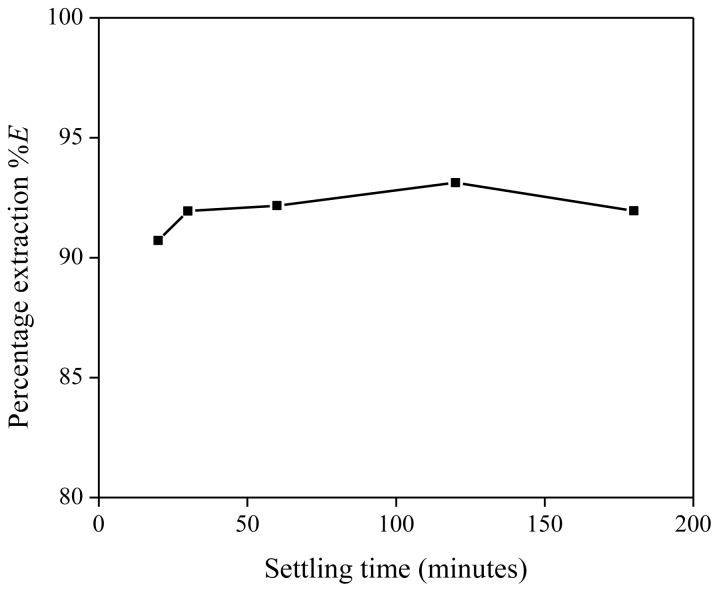
Percentage extraction (%*E*) as a function of the settling time (in minutes) for the extraction of Nd(Tf_2_N)_3_ in [Hbet][Tf_2_N]–H_2_O with betaine as extractant.

**Figure 7:**
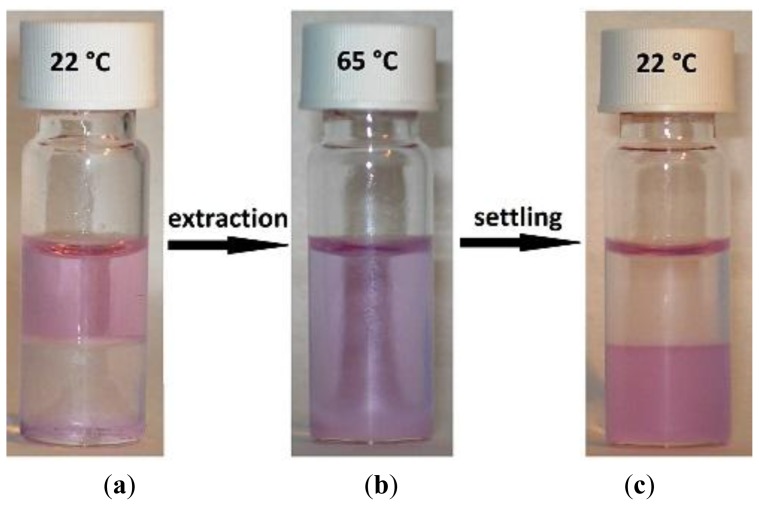
Visual representation of a homogeneous liquid–liquid extraction (HLLE) with [Hbet][Tf_2_N] as organic phase, neodymium as metal and betaine as extractant. (**a**) Water phase brought on top of the IL; (**b**) The mixture above the UCST as a homogeneous phase; and (**c**) The two phases after the extraction.

Besides the influence of the settling temperature on the extraction process, the percentage extraction was also investigated as a function of settling time. The extraction mixture contained 5 g of [Hbet][Tf_2_N], 5 g of Nd(Tf_2_N)_3_ solution (1500 mg/kg Nd) and 636 mg of betaine. At different time intervals during the settling stage, a small sample was taken from both the organic and aqueous phase for analysis. The percentage extraction showed a slight increase with longer settling times (Figure 6). If the settling time was too short, the phase separation had not reached its equilibrium yet and a high amount of organic phase (with the extracted metals) was still present in the aqueous phase and *vice versa*. Both phases had a rather turbid appearance during the settling stage, opposite to the more transparent appearance after phase-equilibrium had been reached. During the settling stage, the metal concentration in the aqueous phase tended to be higher and the metal concentration in the organic phase tended to be lower than at equilibrium. As a result, lower extraction percentages were observed. On the other hand, it has been observed that settling can be significantly accelerated by centrifugation. A visual representation of the extraction is given in Figure 7.

### 2.4. Extraction Mechanism

In order to determine the extraction mechanism, a slope analysis was performed. The extraction mechanism and the corresponding equilibrium constant *K*_ext_ can be written as:

(10) $${\text{Nd}}_{\text{aq}}^{3+}+n.{\text{bet}}_{\text{aq}}+3\cdot {\text{Tf}}_{2}{\text{N}}^{-}+x\cdot {\text{H}}_{2}\text{O}⇌\bar{[\text{Nd}{\left(\text{bet}\right)}_{n}{\left({\text{H}}_{2}\text{O}\right)}_{x}]{\left[{\text{Tf}}_{2}\text{N}\right]}_{3}}$$

(11) $$K_{\text{ext}}\frac{{\left[{\text{Nd}}^{3+}\right]}_{\text{IL}}}{{\left[{\text{Nd}}^{3+}\right]}_{\text{aq}}\cdot {\left[\text{bet}\right]}_{\text{aq}}^{n}\cdot {\left[{\text{Tf}}_{2}\text{N}\right]}_{\text{aq}}^{3}}$$

The bar indicates the species in the organic phase.

The distribution ratio *D*_Nd_ is defined as:

(12) $$D_{\text{Nd}}=\frac{{\left[\text{Nd}\right]}_{\text{IL}}}{{\left[\text{Nd}\right]}_{\text{aq}}}$$

where [Nd]_IL_ indicates the concentration of neodmymium in the ionic liquid phase and [Nd]_aq_ is the concentration of neodymium in the aqueous phase. By substitution of Equation (11) in Equation (12), the following expression Equation (13) for the distribution ratio is obtained:

(13) $$D_{\text{Nd}}=K_{\text{ext}}\cdot {\left[\text{bet}\right]}_{\text{aq}}^{n}\cdot {\left[{\text{Tf}}_{2}\text{N}\right]}_{\text{aq}}^{3}$$

**Figure 8:**
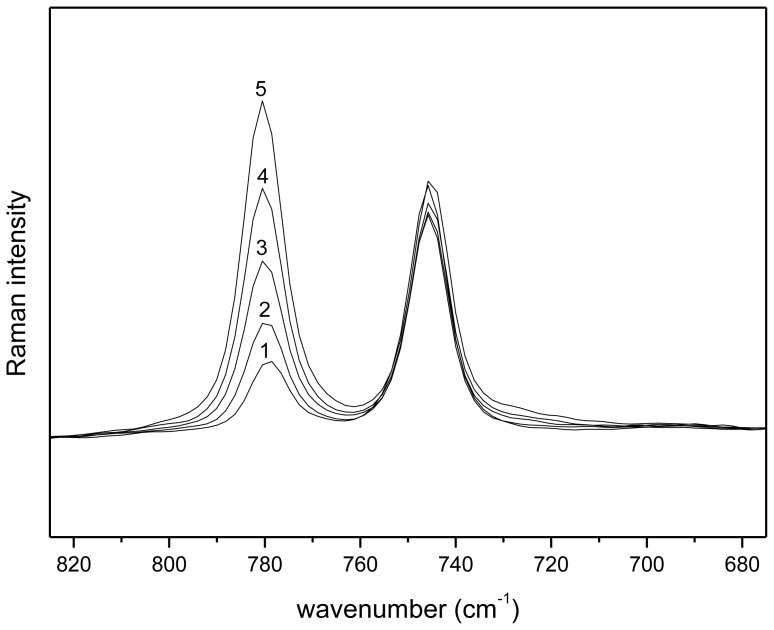
Raman spectra (680–820 cm^−1^) of the aqueous phase of a [Hbet][Tf_2_N]–water two-phase mixture with varying concentrations of betaine: (**1**) 0 wt %; (**2**) 5 wt %; (**3**) 10 wt %; (**4**) 15 wt %; and (**5**) 20 wt % of betaine in the initial aqueous phase. The peak at 780 cm^−1^ corresponds to the γOH bending mode of betaine and the peak at 742 cm^−1^ corresponds to the contraction/expansion of the whole [Tf_2_N] anion [48,49].

By calculating the logarithm of Equation (13), a linear relationship between the logarithm of the distribution ratio and the logarithm of the betaine concentration is obtained. The [Tf_2_N] concentration in the water phase, as a function of the betaine concentration, is considered as a constant value, which was confirmed with Raman spectroscopy (Figure 8). Five aqueous betaine solutions (with concentrations varying between 0 and 20 wt %) were brought in contact with water-saturated [Hbet][Tf_2_N] in a 1:1 mass ratio and mixed until phase equilibrium was reached. The aqueous phase of the mixtures was measured with Raman spectroscopy. By comparison of the peak of betaine at 780 cm^−1^ with the peak of the [Tf_2_N] anion at 742 cm^−1^ in the Raman spectrum, it can be concluded that the [Tf_2_N] concentration in the aqueous phase is almost constant if the betaine concentration in the aqueous phase was increased.

Since also *K*_ext_ is a constant, Equation (13) can be rewritten as:

(14) $$\text{log }D_{\text{Nd}}=\text{constant}+n\cdot \text{log}\left({\left[\text{bet}\right]}_{\text{aq}}\right)$$

**Figure 9:**
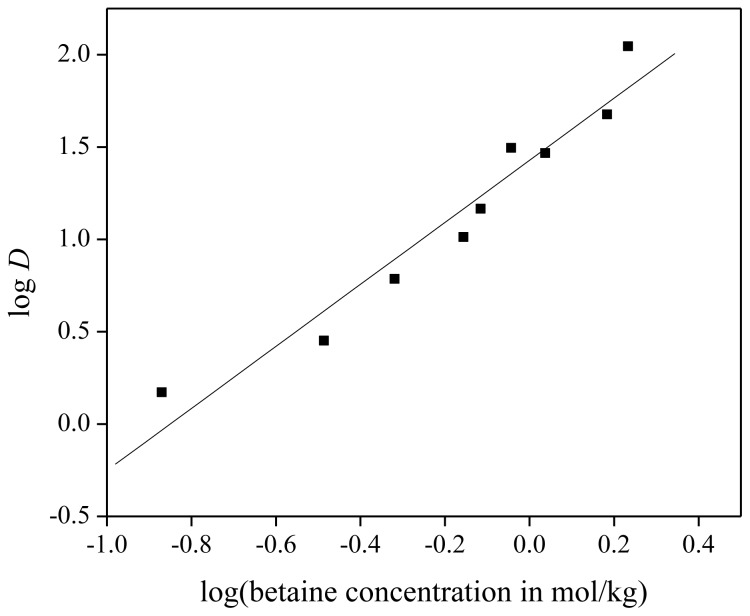
Slope analysis for the extraction of Nd(Tf_2_N)_3_ in [Hbet][Tf_2_N]–H_2_O with zwitterionic betaine as extractant. Slope of linear fit is equal to 1.68 ± 0.16, the *R*-value of the fit is 0.97.

From Equation (14), the number of betaine molecules per neodymium ion can be determined. For the execution of the experiment, extraction mixtures were prepared with 1 g of dry ionic liquid [Hbet][Tf_2_N], 1 g of aqueous Nd(Tf_2_N)_3_ solution (with a neodymium concentration of 1500 mg/kg) and a certain amount of betaine (ranging between 0 and 250 mg), added to the aqueous solution. The homogeneous phase was obtained by heating to 65 °C and two phases were obtained again by cooling down to room temperature. The slope of the linear fit through the experimental data points was 1.68 ± 0.16 (Figure 9). This indicates that probably 1.5 betaine molecules were present as ligands per neodymium ion in the extracted complex. The remaining vacant coordination sites are very likely occupied by water molecules, so that the complex formed during extraction can be described as [Nd_2_(bet)_3_(H_2_O)*_y_*]^3+^. Electrical neutrality can be achieved by bis(trifluoromethylsulfonyl)imide (or chloride or nitrate) counter ions. This is different from what has been reported in the solid state, where binuclear complexes of rare earths, surrounded by eight betaine ligands, are reported. In these cases, the counter anions are always weakly coordinating anions such as [Tf_2_N]^−^ or [ClO_4_]^−^[22,50–52]. A rare-earth metal: betaine ratio of 1:2 has been found as well with chloride anions as counter ions [50,51]. It should be noted that in order to obtain higher distribution ratios, betaine must be present in a large excess compared to the concentration of neodymium(III) ions in the solution (100 or more times a higher concentration of betaine than neodymium). This large excess seems to be required in order to shift the extraction equilibrium [Equation (10)] to the right, *i.e.*, to complex formation and extraction to the organic phase. A stoichiometric amount of betaine was found not to be sufficient for a significant degree of extraction of neodymium to the organic phase.

**Figure 10:**
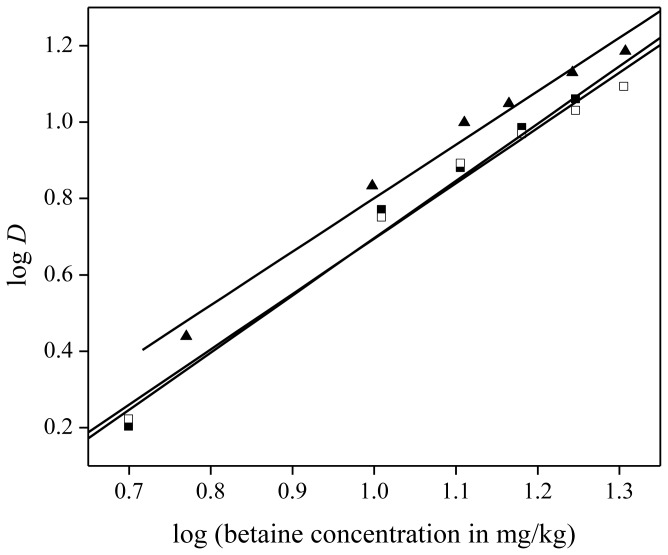
Slope analysis for the extraction of neodymium from chloride (▪), nitrate (□) and bis(trifluoromethylsulfonyl)imide (▴) feed solution in [Hbet][Tf_2_N]–H_2_O with zwitterionic betaine as extractant. The slope of linear fit is equal to 1.50 ± 0.10 (▪), 1.40 ± 0.10 (□), 1.45 ± 0.09 (▴), respectively.

The extraction of NdCl_3_ and Nd(NO_3_)_3_ was tested as well and compared to the extraction of Nd(Tf_2_N)_3_. Feed solutions of neodymium(III) chloride, neodymium(III) nitrate, and neodymium(III) bis(trifluoromethylsulfonyl)imide) were prepared by dissolving the corresponding hydrated salt in distilled water and diluting it further until neodymium concentrations of 1500 mg/kg were obtained. Extraction from all three solutions was tested with varying betaine concentrations in the aqueous feed (5 to 20 wt %). An aliquot of 1 g of each feed solution was mixed with 1 g of water-saturated [Hbet][Tf_2_N]. Extraction from chloride and nitrate medium resulted in similar, but slightly lower distribution ratios compared to extraction from bis(trifluoromethylsulfonyl)imides medium (Figure 10). This deviation may be explained by the influence of different ions present in the [Hbet][Tf_2_N]–H_2_O mixture on the solubility of [Hbet][Tf_2_N] in the aqueous phase. As will be described further in this paper, addition of HCl, and more particularly Cl^−^ ions, results in an increase in cloud point, whereas addition of HTf_2_N (*i.e.*, Tf_2_N^−^ ions) [40] has a lowering effect on the cloud point. This will also influence the solubility of [Hbet][Tf_2_N] in water, with a decrease in solubility for HCl and an increase for HTf_2_N. If more [Hbet][Tf_2_N] dissolves in the aqueous phase, the volume of the organic phase decreases and the concentration of neodymium in the organic phase increases, leading to higher distribution ratios. Another explanation could be the difference in extraction mechanism depending on the anion of the metal salt. The counter ion of neodymium is co-extracted with the metal in the case of a Tf_2_N^−^ anion whereas ion exchange is occurring in the case of a chloride or nitrate counter anion, in which the water soluble ionic liquids [Hbet][Cl] or [Hbet][NO_3_] are formed with the [Hbet]^+^ cation. In all cases, the counter ions of Nd^3+^ in the organic phase are Tf_2_N^−^ anions. This hypothesis was confirmed by extracting 2000 ppm Pr^3+^ with bromide anions to [Hbet][Tf_2_N] with 200 mg of betaine monohydrate. The choice went to a bromide salt because they are more sensitive for X-rays compared to nitrogen, oxygen or chlorine when analyzing concentrations with TXRF. The distribution ratio for Pr^3+^ was 17 whereas the distribution ratio for bromide was 0.03, which confirmed the hypothesis of cation exchange in the case of nitrate, chloride or bromide counter ions.

A slope analysis was performed again to determine the stoichiometry of betaine in the extracted neodymium(III) complex. Extraction from all three media resulted in approximately the same slope of 1.50 and hence the same stoichiometry of betaine in the extracted neodymium(III) complex, namely 1.5 betaine ligands per Nd^3+^ ion (Figure 10). Therefore, the anion of the salt in the feed solution has no influence on the stoichiometry of the neodymium(III)-betaine complex and the anion probably does not interact with the neodymium(III) ion itself.

**Figure 11:**
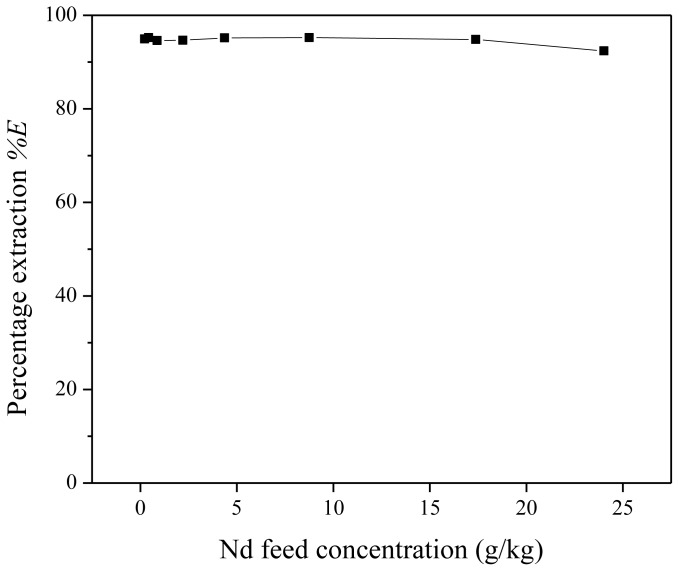
Percentage extraction (%*E*) as a function of the neodymium feed concentration for the extraction of Nd(Tf_2_N)_3_ in water-saturated [Hbet][Tf_2_N] with zwitterionic betaine as the extractant.

Low metal feed concentrations are not of interest for extraction processes on an industrial scale. Therefore, the effect of the feed metal concentration on the extraction performance was studied. Different extraction mixtures were prepared, all containing 1 g of water-saturated [Hbet][Tf_2_N] and 1 g of Nd(Tf_2_N)_3_ feed with a betaine concentration of 200 g/kg. The neodymium concentration in the feed varied between 224 and 24,000 mg/kg. The percentage extraction appears to be rather independent of the neodymium feed concentration (Figure 11). This constant behavior in function of feed concentration can possibly be explained by the presence of an excess of betaine extractant. The extraction mechanism and equilibrium constant were already given in Equations (10) and (11). The factor ${\left[\text{bet}\right]}_{\text{aq}}^{n}\cdot {\left[{\text{Tf}}_{2}\text{N}\right]}_{\text{aq}}^{3}$ in Equation (11) remains almost constant during the extraction because ${\left[\text{bet}\right]}_{\text{aq}}^{n}$ and ${\left[{\text{Tf}}_{2}\text{N}\right]}_{\text{aq}}^{3}$ are present in the aqueous phase in a large excess compared to neodymium in the aqueous phase and neodymium extracted to the ionic liquid phase.

Extraction of neodymium will only slightly decrease the denominator of Equation (11). In this case, Equation (11) can approximately be rewritten as:

(15) $$K^{*}\approx \frac{{\left[{\text{Nd}}^{3+}\right]}_{\text{IL}}}{{\left[{\text{Nd}}^{3+}\right]}_{\text{aq}}}$$

For the above equation, one can deduce that if the concentration of [Nd^3+^]_aq_ increases in the feed solutions, the amount of neodymium extracted to the ionic liquid phase will increase as well, in a similar proportion.

The extraction percentage (%*E*) of neodymium is defined as:

(16) $$\%E=\frac{{\left[\text{Nd}\right]}_{\text{IL}}\cdot m_{\text{IL}}}{{\left[\text{Nd}\right]}_{\text{IL}}\cdot m_{\text{IL}}+{\left[\text{Nd}\right]}_{\text{aq}}\cdot m_{\text{aq}}}$$

In our experiments, 1 g of water-saturated ionic liquid was mixed with 1 g of the aqueous phase (*m*_IL_ = *m*_aq_). Assuming that no volume or mass changes occur, Equation (16) can be rewritten as:

(17) $$\%E=\frac{{\left[{\text{Nd}}^{3+}\right]}_{\text{IL}}}{{\left[{\text{Nd}}^{3+}\right]}_{\text{IL}}+{\left[{\text{Nd}}^{3+}\right]}_{\text{aq}}}$$

Or by introducing Equation (15) and replacing [Nd^3+^]_aq_:

(18) $$\%E=\frac{{\left[{\text{Nd}}^{3+}\right]}_{\text{IL}}}{{\left[{\text{Nd}}^{3+}\right]}_{\text{IL}}+\frac{{\left[{\text{Nd}}^{3+}\right]}_{\text{IL}}}{K^{*}}}=\frac{1}{1+\frac{1}{K^{*}}}$$

This means that the percentage extraction is constant as long as the assumption stating that the Tf_2_N^−^ anion and especially betaine are present in excess holds. It was observed that the phase volume ratio of the organic over aqueous phase increased with increasing neodymium feed concentration. This may also influence the change in calculated percentage extraction *versus* the neodymium feed.

### 2.5. Metal Stripping and Recovery of the Ionic Liquid

**Figure 12:**
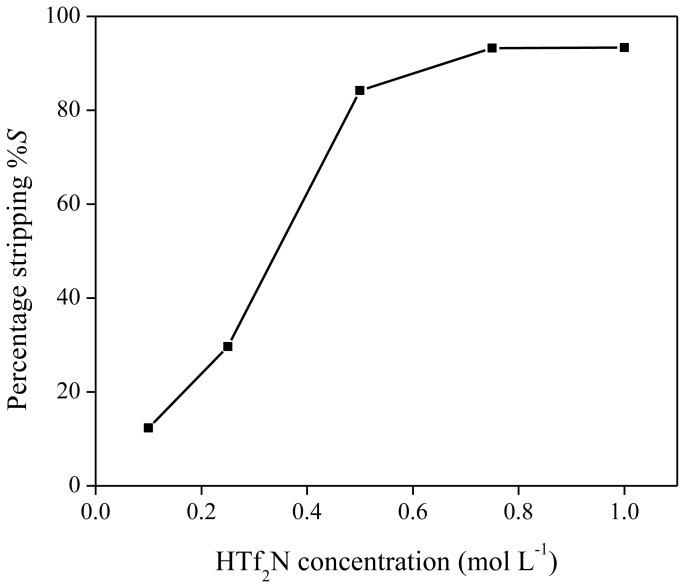
Percentage stripping (%*S*) as a function of different HTf_2_N concentrations for the stripping of neodymium from [Hbet][Tf_2_N].

**Figure 13:**
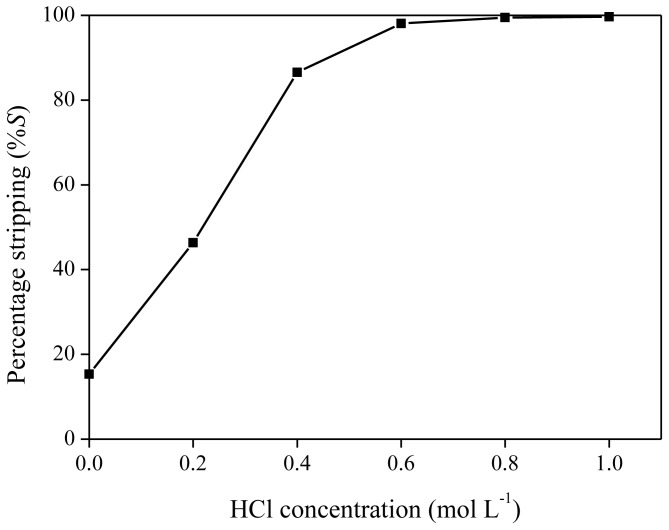
Percentage stripping (%*S*) as a function of different HCl concentrations for the stripping neodymium from [Hbet][Tf_2_N].

As reported previously, neodymium can be stripped from the ionic liquid phase by acids, such as HTf_2_N or HCl [40]. The influence of HTf_2_N on the stripping efficiency was measured by stripping a neodymium-loaded ionic liquid phase obtained by extraction of 6 mL of Nd(Tf_2_N)_3_ solution (1500 mg/kg Nd) with 1.250 g of betaine in 5 g of [Hbet][Tf_2_N]. HTf_2_N stripping solutions (500 μL) with concentrations varying between 0.1 and 1 M were mixed with 500 mg of ionic liquid phase. Stripping was also performed by heating the solution above the UCST (65 °C) for 5 min in order to obtain mixing on a molecular scale. As expected, more concentrated hydrogen bis(trifluoromethylsulfonyl)imide strip solutions resulted in higher stripping efficiencies, with a value of approximately 95% for a 1 M HTf_2_N solution (Figure 12). Therefore, stripping is possible with hydrogen bis(trifluoromethylsulfonyl)imide (1 M) if the extraction mixtures are cooled to 6 °C during the settling stage because no biphasic system could be obtained at higher temperature. Stripping with HTf_2_N is limited due to the mutual solubility of [Hbet][Tf_2_N] and water, which increases at higher acid concentration. By stripping with HTf_2_N, the ionic liquid itself is generated. Hydrogen chloride is an even better stripping agent than HTf_2_N. This was shown by stripping an organic phase of [Hbet][Tf_2_N], loaded with 1500 mg/kg Nd after extraction from a bis(trifluoromethylsulfonyl)imide aqueous solution, with HCl stripping solutions varying between 0 and 1 M. Phase separation was very easily obtained and there was no need for cooling to 6 °C during settling. The cloud point increased with higher HCl concentrations, no homogeneous phase was formed during the heating/mixing stage. This difference in phase behavior compared to HTf_2_N is caused by salting-in/salting-out phenomena and will be discussed further in the text. It was found that stripping efficiencies of 99% could be obtained for HCl concentrations of 0.8 M and higher (Figure 13). Lower acid concentrations were needed than in the case of HTf_2_N to obtain the same stripping percentage. By stripping with HCl, [Hbet]Cl is formed, and the ionic liquid [Hbet][Tf_2_N] can be synthesized again by a metathesis reaction with e.g., LiTf_2_N as described in the experimental part.

The pH of the aqueous phases after extraction was about 0.5 in a 1:1 IL/water mixture at room temperature and pK_a_ of betaine is 1.82 [53]. Therefore, the use of acidic extractant will become problematic as these are protonated and not useful anymore at these pH values.

### 2.6. Influence of Betaine and HCl on the UCST of [Hbet][Tf_2_N]

**Figure 14:**
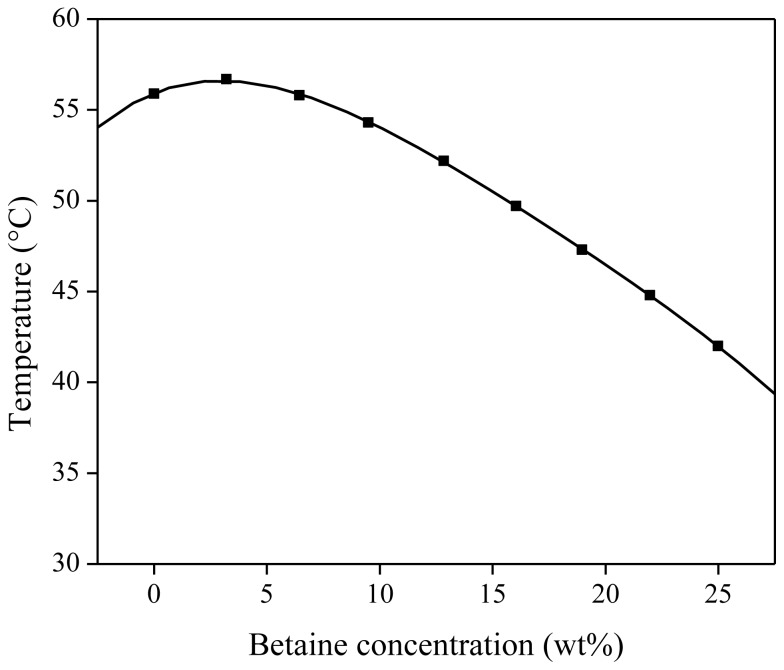
Influence of the betaine concentration in the aqueous phase on the cloud point of a [Hbet][Tf_2_N]–water mixture (50/50 wt % [Hbet][Tf_2_N]_sat_/H_2_O), sat: saturated.

As mentioned above and reported earlier [40], the addition of acids or betaine influences the phase behavior of the ionic liquid in water. The addition of betaine lowers the cloud point curve. The influence of different weight percentages of betaine on the UCST is shown in Figure 11. Mixtures of 500 mg of water-saturated [Hbet][Tf_2_N] and 500 mg of betaine aqueous solution with varying concentration were prepared. The cloud point of each mixture was determined visually and plotted *vs.* the betaine concentration. The addition of betaine to the IL–water mixtures resulted in a significant decrease in cloud point temperature, up to a cloud point of 42 °C for the mixture with 25 wt % betaine in the aqueous starting solution (Figure 14).

**Figure 15:**
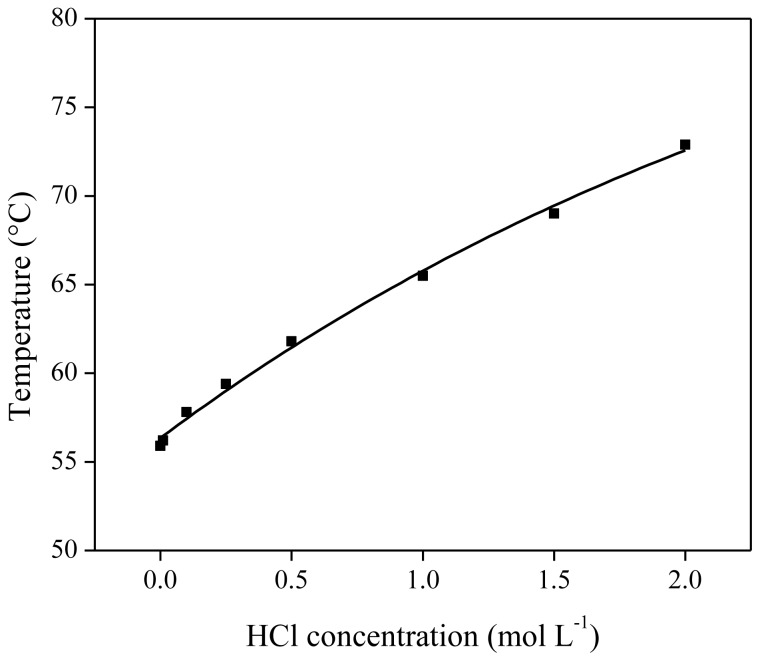
Influence of the HCl concentration in the aqueous phase on the cloud point of the [Hbet][Tf_2_N]–water mixture (50/50 wt % [Hbet][Tf_2_N]_sat_/H_2_O).

As shown above, HCl was found to be a good stripping agent for the stripping of metal loaded [Hbet][Tf_2_N]. To examine the influence of HCl in the stripping solution on the thermomorphic behavior of the [Hbet][Tf_2_N]–water system, the cloud point temperature of a 50/50 wt % [Hbet][Tf_2_N]_sat_–H_2_O mixture was determined as a function of the HCl concentration in the aqueous phase. Ionic liquid mixtures were prepared containing 500 mg of saturated [Hbet][Tf_2_N]_sat_ and 500 mg of diluted HCl solution (concentrations ranging from 0 to 2 mol/L). The observed cloud point temperature of each mixture was plotted *versus* the HCl concentration in the aqueous solution (Figure 15). The cloud point increased significantly with higher HCl concentrations. A possible explanation for this observation is the increase in ionic strength by addition of chloride ions, which results in a lower solubility of the ionic liquid in water (salting-out effect) [31,54]. An increasing water miscibility between water and 1-butyl-3-methylimidazolium bis(trifluoromethylsulfonyl)imide by adding HTf_2_N has been observed as well by Gaillard *et al.* [55]. They also found an increasing water miscibility when HCl was added, which is opposite from what has been observed here for the addition of HCl to [Hbet][Tf_2_N].

### 2.7. Recovery of the Ionic Liquid

One of the disadvantages of a homogeneous liquid–liquid extraction system based on ionic liquids is the high solubility of the ionic liquid in the water phase. In this way, part of the ionic liquid is lost during the extraction and stripping steps. To investigate the recovery of the ionic liquid from both aqueous phases, collected after stripping and after extraction, respectively, a typical extraction and back-extraction cycle was executed with 10.0 g of water-saturated [Hbet][Tf_2_N] (containing approximately 8.8 g of pure [Hbet][Tf_2_N]), 2.0 g of betaine and 8.0 g of La(Tf_2_N)_3_ solution (2300 mg/kg La). The mixture was heated to obtain one homogeneous phase and subsequently cooled to induce phase separation. The organic phase was isolated (9.38 g) and added to 9.38 g of a 2 M HCl stripping solution. This mixture was reheated to obtain one homogeneous phase and cooled to induce phase separation. The organic phase (7.98 g) was dried on a rotary evaporator (80 °C) and dry [Hbet][Tf_2_N] (6.84 g, 77% of the original amount of [Hbet][Tf_2_N]) was obtained. This [Hbet][Tf_2_N] sample was analyzed with TXRF to determine the remaining metal content. The results showed that the ionic liquid contained no significant amounts of chloride impurities and that lanthanum was completely removed.

The collected aqueous phases (after extraction and after stripping) also contained a certain amount of [Hbet][Tf_2_N], since this ionic liquid dissolves in water. By adding an aqueous CaCl_2_ solution (1.2 mol/kg) to both aqueous phases, the solubility of [Hbet][Tf_2_N] decreased and a second phase appeared at the bottom of the vessel. These ionic-liquid-rich phases were isolated, dried on a rotary evaporator (80 °C), and tested with TXRF on their metal content.

The ionic liquid recovered from the aqueous phase after extraction (0.90 g, 10% of the original amount of [Hbet][Tf_2_N]) contained high concentrations of lanthanum and calcium. The ionic liquid recovered from the aqueous phase after stripping (0.42 g, 5% of original amount of [Hbet][Tf_2_N]) also contained calcium and lanthanum, but in far lower concentrations, despite the higher lanthanum concentrations in this aqueous phase. This can be explained by the high HCl stripping concentrations in the solution and the absence of betaine as extractant. In comparison, no HCl but high amounts of betaine are present in the aqueous phase after the extraction, which are optimal extraction conditions. This difference in composition is probably the major reason why the metal concentrations in the recovered ionic liquid phases are different. Lanthanum impurities could be completely removed from both parts of the recovered [Hbet][Tf_2_N] by increasing the HCl concentration in the corresponding aqueous solution up to 4 M. The calcium concentration was only lowered to approximately 100 mg/kg. Thus, a second purification step of stripping with HCl is required to completely remove all calcium from [Hbet][Tf_2_N] recovered from the aqueous phases. The total recovery percentage of [Hbet][Tf_2_N] was 92%.

## 3. Experimental Section

### 3.1. Products

Anhydrous betaine (98%), neodymium(III) nitrate hexahydrate (99.9%), neodymium(III) oxide (99.99%) and neodymium(III) chloride (99.9%), praseodymium(III) bromide (99.9%) were purchased from Alfa Aeser (Karlsruhe, Germany). Lithium bis(trifluoromethylsulfonyl)imide (99%) and hydrogen bis(trifluoromethylsulfonyl)imide (99%) were obtained from IoLiTec (Heilbronn, Germany). Betaine monohydrate (99+%) and betaine chloride (99%) were from ACROS Organics (Geel, Belgium). Absolute ethanol and hydrogen chloride (37%) were obtained from VWR (Leuven, Belgium). A 1000 ppm gallium and dysprosium standard were obtained from Merck (Overijse, Belgium). Calcium chloride (99%) was purchased from Sigma-Aldrich (Diegem, Belgium). The silicone solution in isopropanol was obtained from SERVA Electrophoresis GmbH (Heidelberg, Germany).

### 3.2. Equipment

^1^H- and ^13^C-NMR spectra were recorded on the Bruker Avance 300 spectrometer (Bruker Biospin, Rheinstetten, Germany). A frequency of 300 MHz was used for recording the ^1^H spectra and 75 MHz for the ^13^C spectra. The samples were prepared by dissolving a small amount of product in deuterated dimethyl sulfoxide ([D_6_]DMSO) or deuterated water (D_2_O). Melting points of the synthesized ionic liquids were recorded on a power compensation DSC (DSC 882^e^, Mettler-Toledo, Greifensee, Switzerland) in a helium atmosphere. Densities were measured with a 5 mL pycnometer. Elemental analysis (carbon, hydrogen, nitrogen) was performed on a CE Instruments EA-1110 element analyzer (Interscience, Louvain-la-Neuve, Belgium). FTIR spectra were recorded on a Bruker Vertex 70 spectrometer and analyzed with OPUS software (Bruker Optics, Ettlingen, Germany). Both solid and liquid samples are examined directly without further preparation using a Platinum ATR single reflection diamond attenuated total reflection (ATR) accessory (Bruker Optics, Ettlingen, Germany). A Mettler-Toledo DL39 coulometric Karl Fischer titrator (Mettler-Toledo, Greifensee, Switzerland) was used to determine the water content in the synthesized ionic liquids. Raman spectra of the aqueous phases were recorded on a Bruker Vertex 70 spectrometer with a Ram II Raman module (Bruker Optics, Ettlingen, Germany), with 128 scans per spectrum at a laser power of 500 mW and a resolution of 4 cm^−1^.

### 3.3. Synthesis

#### 3.3.1. Synthesis of [Hbet][Tf_2_N]

Betaine hydrochloride (0.195 mol, 30.005 g) and lithium bis(trifluoromethylsulfonyl)imide (0.196 mol, 56.200 g) were added to 50 mL of water and stirred for one hour. The resulting ionic liquid phase-separated from the aqueous layer. Lithium chloride, formed during the reaction, dissolved mostly in the aqueous phase. The ionic liquid was washed with ice-cold water to remove chloride impurities. The presence or absence of chloride impurities was tested by addition of silver nitrate to the aqueous phase obtained after a washing step. Finally, the remaining water in the ionic liquid was removed *in vacuo* on a rotary evaporator (80 °C) and the compound was obtained as a white solid product. Yield 80% (0.156 mol, 62.2 g). ^1^H-NMR (300 MHz, D_2_O, δ/ppm): 4.12 (s, 2H, CH_2_), 3.21 (s, 9H, 3 × CH_3_). ^13^C-NMR (75.47 MHz, D_2_O, δ/ppm): 167.06 (COO), 121.35 (q, 2 × CF_3_, *J* = 4.24), 63.71 (N–CH_2_), 53.77 (3 × CH_3_). Elemental analysis calculated for C_7_H_12_N_2_O_6_F_6_S_2_ (M = 398.302 g/mol) (%): C 21.11, H 3.04, N 7.03; found (%): C 21.65, H 2.53, N 6.95. Water content: 0.16 wt % (1568 ppm). Density: 1.541 g·cm^−3^ (at 60 °C). Melting point: 58.6 °C. FTIR (ATR, cm^−1^) 1768 (COOH), 1478 (CH_2_), 1420, 1348 (SO_2_), 1325 (SO_2_), 1180 (CF_3_), 1137 (SO_2_), 1050 (SO_2_), 741 (CF_3_), 570 (CF_3_), 513 (CF_3_).

#### 3.3.2. Synthesis of Metal Bis(trifluoromethylsulfonyl)imides

Neodymium(III) oxide (0.005 mol, 1.676 g) was added to a solution of 80 wt % hydrogen bis(trifluoromethylsulfonyl)imide (0.030 mol, 10.56 g) in 50 mL of water and lanthanum(III) oxide (0.005 mol, 1.614 g) was added to a solution of 80 wt % hydrogen bis(trifluoromethylsulfonyl)imide (0.030 mol, 10.56 g) and dissolved as well in 50 mL of water. Both solutions were stirred under reflux conditions for one hour. The reaction mixtures turned slowly into a clear solution after approximately 30 min. Reaction was followed by measuring the pH. The product was dried *in vacuo* at a Schlenk line to remove remaining water.

### 3.4. Extraction Experiments

During a typical extraction experiment, 1 g of water saturated ionic liquid was mixed in a 1:1 mass ratio with an aqueous solution of metal ions and the betaine extractant. The choice for using mass ratios instead of the commonly used volume ratios was made because weighing the ionic liquid is easier and more precise than pipetting a viscous ionic liquid. The mixture was heated above the UCST and gently shaken with a Nemus Life Thermo Shaker TMS-200 (Nemus Life AB, Lund, Sweden) to obtain one phase (homogeneous stage). Gentle agitation was applied to speed up the formation of one phase, because no homogeneous solution is formed without shaking in the time span of the extraction process at temperatures slightly above the UCST. This is due to the large difference in density between the water phase and the ionic liquid phase. Next, the mixture was cooled to room temperature (settling stage) to return to a two-phase equilibrium and the settling process was accelerated by centrifugation. The metal content was measured in both phases with a Bruker S2 Picofox Total Reflection X-Ray Fluorescence (TXRF) spectrometer (Bruker AXS Microanalysis GmbH, Berlin, Germany). A known amount of gallium or dysprosium nitrate was added as internal standard to a small amount of the solution (typically 100 mg). The mixture was further diluted up to approximately 1 g with Milli-Q^®^ water (Billerica, MA, USA) for aqueous samples and with ethanol for ionic liquid samples and homogenized using a vortex mixer. A small droplet of the prepared sample solution (5–10 μL) was dispensed on a quartz sample carrier and dried in the oven at 60 °C for 15 min to remove volatile solvents. To prevent droplets of aqueous solutions to spread out or roll to the side of the carrier, a small amount of SERVA silicone solution in isopropanol (20 μL) was dispensed on the quartz carrier and dried at 60 °C for 15 min prior to addition of the sample solution droplet.

### 3.5. Cloud Point Determinations

The following experimental set-up is used for the determination of the cloud points: a solvent mixture is placed in a small test tube (diameter: 10 mm, length: 13 mm) and stirred with a magnetic stirring bar. The mixture is placed in a temperature-controlled water (or silicone oil) bath under constant stirring and heated until phase separation disappears. The water bath was then allowed to cool gradually (maximum cooling speed of 0.6 °C/min) and the transparency of the mixtures was monitored by sight to determine the temperature at which the mixture became cloudy (cloud point).

### 3.6. Viscosity Measurements

Viscosity was measured with a falling-ball viscosimeter size number three (Gilmont Instruments, Barrington, IL, USA) and a glass or stainless steel ball. A 4 g sample of water-saturated ionic liquid ([Hbet][Tf_2_N]_sat_) was inserted into the viscosimeter. A 3.48 g sample of water containing 20 wt % of betaine was added to obtain a biphasic mixture with a 47/53 wt % water/[Hbet][Tf_2_N]_sat_ composition similar as in the extraction experiments. The viscosimeter was closed and heated until 75 °C in a water bath. Afterwards, the mixture was manually shaken to obtain one phase, reinserted in the water bath and cooled down for two hours until the desired temperature was obtained. A stainless steel ball and a distance of descent of 6 cm were used for the temperatures under the UCST. The glass ball was used for the low viscous mixtures above the UCST and the distance of descent was, in that case, 10 cm. The viscosity was calculated by using the following formula:

(19) $$\eta =K\cdot (\rho_{\text{b}}-\rho_{\text{IL}})\cdot t$$

Here, η is the viscosity, K is a constant (35 mPa·cm^3^·g^−1^) and specific for the applied viscosimeter, *ρ*_b_ is the density of the ball (*ρ*_b_ = 2.53 g·cm^−3^ for the glass ball and 8.02 g·cm^−3^ for the stainless steel ball), *ρ*_IL_ is the density of the ionic liquid, and *t* is the time necessary for the descent. The density of the water saturated ionic liquid at room temperature is 1.466 g·L^−1^. Although this value will slightly change at higher temperature due to thermal expansion and a change in composition, it was used for all measurements under the UCST. The use of a constant value is justified because the density of the ball (8.02 g·cm^−3^) is significantly higher than the density of the ionic liquid. The error made by using a constant value is estimated to be below 4%. A small error was made as well for the measurements above the UCST because a constant density value of 1.242 g·mL^−1^ was used, which is the calculated density of a 47/53 wt % water/[Hbet][Tf_2_N]_sat_ mixture at 60 °C without the addition of betaine.

## 4. Conclusions

The ionic liquid [Hbet][Tf_2_N] with an UCST of 55 °C, in combination with betaine as extractant, has been used as a homogeneous liquid–liquid extraction system for neodymium. The influences of several operational parameters on the extraction were investigated: heating time, duration of the homogeneous stage, temperature of the homogeneous stage, settling time and temperature of the settling stage. Heating for 2 min just above the UCST (60 °C) and cooling down to room temperature seems to be long enough to reach equilibrium with a minimum of energy input. It was confirmed that the homogeneous stage in the extraction process has a positive influence on the extraction kinetics. The metal feed concentration had no influence on the distribution ratio and the solubility of the metal complex did not hinder extraction. Extraction is not only possible from bis(trifluoromethylsulfonyl)imide medium, but also from chloride and nitrate media, with similar distribution ratios. The extraction mechanism of neodymium was investigated by slope analysis and it was found that a complex with a betaine:neodymium ratio of 1.5 was formed. Back-extraction (stripping) was possible with acidic aqueous solutions of hydrogen bis(trifluoromethylsulfonyl)imide and hydrogen chloride. The best results were obtained for hydrogen chloride. After the back-extraction step, the ionic liquid [Hbet][Tf_2_N] did not contain any impurities and could be reused without further purification. The ionic liquid dissolved in the aqueous phases during extraction and back-extraction could be partially recovered by addition of CaCl_2_ as a salting-out agent. A drawback of this recovering process is contamination of the recovered [Hbet][Tf_2_N] by Ca^2+^ ions. However, this contamination could be minimized by the addition of HCl to the aqueous phase, together with the CaCl_2_ salt. The phase behavior of the [Hbet][Tf_2_N]–water solvent system and the influence of additives such as betaine and HCl were examined. Extraction equilibrium is obtained faster by increasing the temperature, reducing the viscosity and thus increasing the diffusion of ions, by performing the extraction above the UCST and performing the extraction on a molecular scale without the presence of phase boundaries. Moreover, the energy barrier for movement of the ionic liquid molecules along each other is 2.5 times lower above than below the UCST.
